# Brazilian disaster datasets and real-world instances for optimization and machine learning

**DOI:** 10.1016/j.dib.2022.108012

**Published:** 2022-03-05

**Authors:** Rafaela Veloso, Juliana Cespedes, Aakil Caunhye, Douglas Alem

**Affiliations:** a Aeronautics Institute of Technology, São José dos Campos-SP, Brazil; b Business School, The University of Edinburgh, Edinburgh, United Kingdom; c Institute of Science and Technology, Federal University of São Paulo, Séo Joé dos Campos-SP, Brazil

**Keywords:** Disaster management, Emergency response, Humanitarian logistics, Disaster impact data, Socioeconomic and geographical data, Machine learning, Optimization instances and algorithms

## Abstract

We present comprehensive datasets of Brazilian disasters from January 2003 to February 2021 as well as real-world optimization instances built up from these data. The data were gathered through a series of open available reports obtained from different government and institutional sources. Afterwards, data consolidation and summarization were carried out using Excel and Python. The datasets include 9 types of disaster, such as flash floods, landslides and droughts, and the corresponding number of affected people during an 18-year or a 218-month observation period for 5,402 Brazilian municipalities, totaling more than 65,000 observations. Data on relevant geographical, demographic and socioeconomic aspects of the affected municipalities are also provided. These encompass geographic coordinates, regions, population, income, development indicators, amongst other information. From a statistical point of view, the data on disasters can address a number of applications using both supervised and unsupervised machine learning techniques such as, for time series analysis or other dynamic models using socioeconomic data as explanatory variables, i.e. data on the size of the poor population, income, education and general development. The geographic dataset can be useful for aggregating analyses concerning the various forms of territorial organization and allows for the visualization of data in maps. All the aforementioned data can be also used to devise realistic optimization instances related to diverse humanitarian logistics and/or disaster management problems, such as facility location, location-allocation, vehicle routing, and so forth. In particular, we describe two real-world instances for the location-allocation problem studied in [1]. For that purpose, we partially use the given datasets and included other information such as costs and distances relevant to the optimization model. Although using real-world cases to test optimization approaches is a common and encouraged practice in Operations Research, comprehensive datasets and practical optimization instances, as presented in this article, are rarely described and/or available in the academic literature.

**Specifications Table**

**Table:** 

Subject	Management Science and Operations Research
Specific subject area	Disaster Management, Humanitarian Logistics, Relief-aid Planning, Supply Chain Design.
Type of data	Tables; R code.
How data were acquired	The data were collected from a series of open data sources and documents.
Data format	Analyzed; Consolidated; Filtered.
Parameters for data collection	Data consolidation and summarizing from the original reports and other acquisition formats were necessary. Some of the data were modified according to specific assumptions to obtain the optimization instances.
Description of data collection	The data were mainly manually collected from public sources and official government data portals. Manually collected data include electronic spreadsheets, PDF reports (.pdf), and text information. We had also to generate a number of reports from their respective primary sources, one by one, due to limitations of time periods and other parameters options for data extraction. An extensive research was made necessary to obtain missing data and/or data that were not available in a structured format, e.g. for the opening cost parameter - see Section 2.2.3 for further details - we had to access different CUB reports in pdf format for each Brazilian state, and then we managed to compute the respective cost of industrial shed construction in the tabular form. We obtained part of the data from a series of articles available in the literature, and so on. In addition, the distances between locations were gathered through the Openrouteservice package using R. After consolidating the raw data obtained from each of the primary data sources, we filtered only the columns under the scope of this paper, treated duplicates, and consolidated all data in an unique file named ”Brazilian disaster datasets.xlsx” (available on: https://doi.org/10.17632/spzgprdjgj.3). Part of this dataset was transformed and combined to other key data to create realistic optimization instances for humanitarian logistics problems. Both the content of the datasets and the method used to create the instances are detailed described in this article.
Data source location	Country: Brazil. The sources are mainly reports, tables, public notices, etc. from an extensive list of primary data sources*. However, a large portion of the data were collected from: Integrated Disaster Information System (S2ID) [2] Brazilian Institute of Geography and Statistics (IBGE) [3] Atlas of Human Development [4] *The list of all primary data sources are compiled in Metadata.xlsx and are available on: https://doi.org/10.17632/spzgprdjgj.3
Data accessibility	Veloso, R., Cespedes, J., Caunhye, A., Alem, D. (2022), Brazilian disaster datasets and real-world instances for optimization and machine learning, Mendeley Data, V3. Repository name: Mendeley Repository Direct URL to data: https://doi.org/10.17632/spzgprdjgj.3
Related research article	N.A.

## Value of the Data

• The datasets can be used to visualize and analyze the impacts of Brazilian disasters over time and across geographical areas as well as to investigate their links to regional socioeconomic factors. The geographical areas are tagged so that analyses can be performed with different levels of granularity, from region-level, i.e. South, North, Midwest, Northeast and Southeast, to municipality-level.
• The datasets are relevant for researchers to validate predictive analytics techniques. It can be viewed as both cross-sectional and time series data, making it amenable to a wide array of supervised and unsupervised machine learning techniques, from time series analysis to factor analysis, tree-based methods, and clustering. These techniques can be used to predict future impacts based on past impacts and socioeconomic factors to find low-dimensional representation of the relationships between impact, time period, geographical location, and socioeconomic indicators and to identify prevalent or unsuspected trends.
• The timestamped datasets can be used within a wide array of optimization techniques such as to construct scenarios for stochastic programming models, to draw descriptive statistics for robust or distributionally robust optimization, and to create transition states in dynamic programming models. In addition, the data is formatted in a way that it can be easily input into different modeling and optimization software.
• The real-world optimization instances can be directly used for benchmarking purposes for the development of new exact and heuristic methods, to compare existing optimization approaches, and so forth. Moreover, the datasets and/or instances can be used to build other realistic case studies and/or optimization instances, which is recognized to be a missing aspect of several research papers within humanitarian logistics [5], [6]. These optimization instances can be used in a large number of Operations Research applications, such as facility location, resource allocation, location-allocation, last-mile distribution, traveling salesperson and vehicle routing problems, to name a few.
• The secondary data provided within this article refers to a research problem that cannot be addressed with the merely compilation of the primary data alone. We curated data and made assumptions based on our technical knowledge of different disciplines to produce optimization datasets for the specific purpose of supporting analysis and development of ML and optimization models for humanitarian logistics and disaster management, which adds originality to the secondary data, compared to the primary datasets.

## 1 Data Description

The datasets consist of data tables containing information related to the occurrences of disasters in the period 2003-2021 in Brazil, as well as geographic and socioeconomic data. We also provide two real-world based instances for optimization problems in humanitarian logistics (hereafter referred to as HL1 and HL2) partially based on these datasets and, finally, a metadata table containing the description and source details of the instances’ parameters and sets. All data are presented in electronic spreadsheets in the repository https://doi.org/10.17632/spzgprdjgj.3. In addition, we included the R code used to generate the distance matrix, which corresponds to one of the parameters of the provided optimization instances. The instances are organized in a way that the parameters of the model can be easily imported into any optimization software as input. A brief description of each dataset is given first, followed by the description of the optimization instances.

### 1.1 Datasets

• The dataset on Brazilian disasters - worksheet “Disaster data” in file “Brazilian disaster datasets.xls” - contains the numbers of affected people (deceased, injured, sick, displaced, homeless, and others) in Brazil for each month by municipality. It also presents the state each municipality belongs to, its abbreviation, and its corresponding COBRADE^1^, followed by the description of the type of the disaster occurrence. All this information is related to the following types of disasters: drought, flash flood, flood, inundation, landslide, hail storm, heavy rainfalls, gale storm, subsidence, and collapses. The number of victims and occurrences can be aggregated at different geographic levels for further analysis. For that purpose, we have made the geographical data on the municipalities also available.
• The geographical data - worksheet “Geographical data” in file “Brazilian disaster datasets.xls” - presents the municipality, microregion^2^, mesoregion^3^, state, state abbreviation, region, and its respective official code in addition to the geographic coordinates for map visualization and the respective population of each municipality.
• The socioeconomic data - worksheet “Socioeconomic data” in file “Brazilian disas- ter datasets.xls” - presents several socioeconomic indicators, such as income and size of poor populations, and the IDH-M along with its respective components, i.e. income IDH-M, education IDH-M and longevity IDH-M. The Municipal Human Development Index (IDH-M) is a national index based on the Human Development Index (HDI) methodology [7]. According to Pinto et al. [8], the Municipal Human Development Index (IDH-M) was co-developed by the United Nations Development Programme, the Institute of Applied Economic Research (IPEA), and the Jo£o Pinheiro foundation as an adapted methodology to calculate the level of human development of Brazilian municipalities. Similar to the HDI, the IDH-M is a number that varies between 0 and 1. The closer the IDH-M is to 1, the greater the development level of the municipality is. Both the income and size of poor populations and the IDH-M indicators are available for the years of 2000 and 2010, which correspond to the latest data at the municipality level based on the most recent censuses carried out in the country. The socioeconomic data on the number and income of poor populations are grouped into extremely poor, very poor, and poor people. A detailed definition of each group of poor people is available in the metadata table.

### 1.2 Optimization instances

**Fig. 1:**
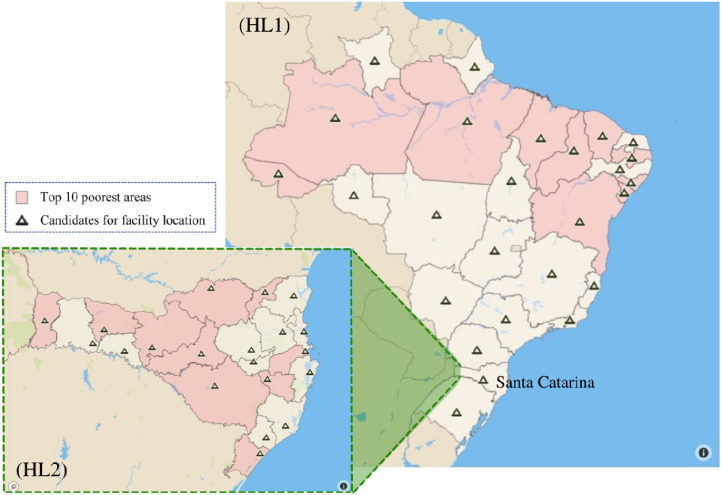
Geographical scope covered by instances HL1 and HL2.

This dataset contains two different optimization instances available in files “HL1.xls” and “HL2.xls” based on a scenario-based two-stage stochastic location-allocation problem [1]. The first instance (HL1) refers to the design of a humanitarian supply chain at the *national* level, where the number of victims (and their needs) in each scenario and the facility location candidates are given for each Brazilian state in an aggregate way. The main decision-maker in this case is the Brazilian Federal Government, via the Ministry of Regional Development. On the other hand, the second instance (HL2) presents data for the same problem, but from a *state* level perspective, in which the aggregate number of victims (and their needs) as well as the facility location candidates were disaggregated at the level of microregions. This was done for the state of Santa Catarina in Brazil, where the state civil defense is responsible for disaster prevention and preparedness in coordination with the State Government of Santa Catarina. Fig. 1 shows the geographical scope of the optimization instances HL1 and HL2, in which we can clearly see that Santa Catarina exhibits only one candidate for facility location in the aggregated instance HL1. On the other hand, the same state has several candidates for facility location in the disaggregated instance HL2.

The instance HL2 differs from HL1 both in terms of the geographical scope of the covered areas, the number of victims and their socioeconomic context, as well as decision-makers. However, it was possible to obtain HL2 combining some of the data from the first instance (HL1) and the Santa Catarina’s data from the provided dataset (“Brazilian disaster dataset.xlsx”). Such procedure can be used to generate many other instances, as further explained at the end of Section 2.

The main optimization problem related to such instances consists of finding the optimal location for service provision (e.g., humanitarian assistance such as relief aid items) across a municipality or region given the spatial distribution of demand (or victim needs) for that service. Therefore, the model will locate a number of relief facilities (relief centers, depots/warehouses, shelters, etc.) and allocate demand zones to each established relief facility (RF). In humanitarian logistics applications, location decisions are usually made in the preparedness phase (before disaster strikes) whereas allocation decisions are often made in the response phase (after disaster strikes). The objective of such problems usually relies on minimizing logistics costs or maximizing service provision. Variations of the problem include equity and vulnerability issues based on geographical and/or socioeconomic data.

Such problems are formulated as mathematical models composed by the objective function and a set of constraints. The constraints are equations that combine the decision variables and known parameters, which are usually related to a set domain. The optimization instance data consist of parameters and set elements. Table 1 shows the information about the input data of both optimization instances (HL1 and HL2). Section 2 will detail each of these optimization instances. New realistic instances can be obtained from a combination between some of the parameters of the optimization instance (HL1) and information from the disaster datasets. The comparison between HL1 and HL2 will clarify this procedure in the next section.

**Table 1:** Parameters of the instances.

Notation	Parameter (Code)	Unit	Description
L,S,R	L, S, R	-	Sets of RF’s sizes, scenarios and types of relief aid items
F	A	-	Set of poverty indicators
N	N, M	-	Set of nodes and respective alias
l,s,r,a	-	-	Elements of sets L,S,R and F, respectively
m,n	-	-	Elements of set N
$d_{rn}^{s}$	D	unt.	Demand for relief aid items at node n in scenario s
lenght	LENGHT	days	Number of days over which the aid need to be available
$coverage_{r}$	COVERAGE	-	Number of people covered by one unit of relief aid r
${people}_{n}^{s}$	VICTIMS	-	Number of victims at node n in scenario s
$l_{r}$	R_DAYS	days	Number of days that the relief aid r lasts
$\pi^{s}$	PI	decimal	Probability of scenario s
${dist}_{mn}$	DISTANCE	km	Distance between nodes m and n
$\kappa_{\ell }^{resp}$	CAP_FAC	m3	Capacity of the RF of size l
$c_{\ell n}^{o}$	Z_COST	BRL (R$)	Opening cost of the RF of size l at node n
$c_{mn}^{\text{s}}$	COST_KM	BRL (R$)/km	Shipping costs from node m to n
α	LOAD	m3	Truck capacity
dieselcost	FUEL	BRL (R$)/l	Average diesel type B S10 cost
consumption	EFFICIENCY	km/l	Fuel economy
$c_{mn}^{d}$	X_COST	BRL (R$)	Round trip cost between nodes m and n
$\rho_{r}$	RHO	m3	Volume of relief aid r
$c_{rn}^{p}$	P_COST	BRL (R$)	Prepositioning cost for relief aid r at node n
$b_{n}^{a}$	P_INDICATOR	% or BRL (R$)	Poverty indicator a at node n
$c_{r}^{h}$	H_COST	BRL (R$)	Holding cost for relief aid r
-	REFERENCE	BRL (R$)	Ref. income: minimum wage in Brazil
η	BUD_1	BRL (R$)	Pre-disaster emergency relief funds
$\eta^{\prime }$	BUD_2	BRL (R$)	Post-disaster emergency relief funds

Notation in accordance to the optimization model developed in [1].

## 2 Experimental Design, Materials and Methods

We gathered data from several sources. Some data were subsequently combined to generate the optimization instances. Most of the data were manually obtained, with the exception of the distances between cities. All the data transformation, such as aggregation and filtering, were done using a spreadsheet software.

### 2.1 Datasets

The data table containing the number of disaster victims in Brazil was obtained from the S2ID platform [2]. All the boxes of the states and the disaster types specified in Section 1.1 were checked. The resulting report is an .xls file with 53 columns for each year which contains information on the numbers of affected people. It must be highlighted that this database is manually fed by municipal authorities who are responsible for the correctness of data. We kept the data that was consistent among all municipalities and we discarded all the other columns. It is important to note that the current data available on the platform only covers the period after 2013. The data on disasters from 2003 to 2012 were obtained by the authors in 2018, when it was possible to obtain older data from the same platform. For that reason, we aggregated the disasters in a monthly basis to make the latest data consistent with our previous dataset.

We obtained the file containing the municipalities’ geographical data from the municipal mesh provided by the IBGE platform [3]. We exported the data contained in the shapefile as a spreadsheet and filtered the columns of interest. The total population of each municipality was also obtained from IBGE [9] and added to the geographical dataset.

Finally, the socioeconomic data were extracted from the Human Development Atlas [4]. The Atlas is published by the United Nations Development Programme (UNDP) in partnership with the Institute of Applied Economic Research (Ipea) and the Jo£o Pinheiro Foundation.

### 2.2 Optimization instances

We partially used the aforementioned datasets to build the optimization instances. All the sources, procedures, and assumptions used to obtain the parameters are described as follow. In addition, the summarized metadata (file “Metadata.xls”) containing the respective sources for each parameter are also provided within the repository related to this article.

#### 2.2.1 Demand or victim needs

We adopted the sum of the number of homeless and displaced people to account for the overall number of victims. Since the disaster reports are organized in a monthly basis, the data of the reports 2003-2021 were consolidated in a single table containing only the total number of victims (displaced and homeless people) for each year. The missing data were substituted by zero to allow for visualization and analytics. We used the aggregated number of victims by state as the victims’ parameter in the optimization instance (HL1) and the number of victims by each microregion of the state of Santa Catarina (SC) as the victims’ parameter in the optimization instance (HL2). The demand represents the victims’ needs in terms of type and quantity of relief aid r for each potential affected area, represented by node n, whose mathematical expression is given by Eq. (1), as follows:

(1) $$d_{rn}^{s}=\left\lceil \frac{lenght}{l_{r}\times coverage_{r}}\right\rceil \times \left\lceil {\text{people}}_{n}^{s}\right\rceil ,$$

assuming that disaster victims require six types of relief aid items: food, water, personnel hygiene, cleaning kits, dormitory kit, and mattress. Here, lenght is the number of days over which victims need to be supplied at node n; $l_{r}$ shows for how long one unit of aid r lasts, and $coverage_{r}$ is the number of people covered by one unit of relief aid r; ‘${\text{people}}_{n}^{s}$’ is the number of people affected by disasters in each Brazilian state n in scenario s. We considered an equal probability of occurrence $\pi^{s}$ for all scenarios in the presented optimization instances (HL1 and HL2).

It is important to note that the parameter lenght is very context-dependent. It may depend on several factors, such as (i) the type and magnitude of the disaster; (ii) the number of days of the response phase; (iii) how fast displaced people will get back to their houses; (iv) the availability of relief aid goods, among others. For this reason, it is well-accepted in the humanitarian logistics literature that typical values vary from some days to a month [10]. Here we considered a seven-day relief response based on the reality of Brazilian disasters whose response phase usually lasts seven days, as claimed in [1]. The seven-day first response phase is also endorsed by several other authors, e.g., [11], [12], [13] and references therein.

#### 2.2.2 Distances

The distance ${\text{dist}}_{mn}$ in km between two locations was obtained via a distance matrix generated by the *Openrouteservice*. This service is useful to compute many-to-many distances and it is based on the data of the *OpenStreetMap* [14], an open initiative to create and provide free geographic data. For the calculation of distances between states we considered the respective capital locations, as they offer a good infrastructure for the potential location of relief-aid facilities. The R code used to obtain the distance matrix generated via the *Openrouteservice* is included in this article data repository. The input file for this application contains the following columns: name of location, longitude and latitude. Such information is available in the presented datasets, but we also included the file coordinates_input.csv in the repository to provide the exact input needed for the distance matrix calculation.

Primary to the service request, it is necessary to obtain a token at the *Openrouteservice* (https://openrouteservice.org/) to get the permissions for the R package usage. We then converted the geographic coordinates in the input file to a list of vectors, which is the adequate format to compute the distance matrix. Detailed steps on the implementation are given as comments within the provided R code. In addition to the distance matrix, the provided code also generates a travel duration matrix, which we did not use in the presented instances, but it can be suitable for other research problems.

#### 2.2.3 Costs

The cost for establishing the RF size ℓ ($c_{\ell n}^{\text{o}}$) was assumed to be proportional to the construction cost given in $m^{2}$. For the Brazilian case, such costs are based on the Basic Unit Costs (CUB) published on monthly reports by the local Unions of Building Construction Industry, which are organized across the states [15]. The Basic Unit Costs are adopted in their respective jurisdictional regions and they are calculated following the criteria and standards of the Brazilian Association of Technical Standards. The publication of CUB reports are mandatory according to Federal Law 4.591 of December 16, 1964. These reports provide a reference of real estate costs and comprise different standards of building construction projects. We adopt the Industrial Shed Basic Unit Cost (GI)^4^ of each state as the proportional cost given in $m^{2}$ to establish the response facility in the respective node.

Shipping costs ($c_{mnk}^{\text{s}}$) were evaluated based on the assumption that relief aid is transported via medium-sized trucks with a capacity of 24 $m^{3}$. These vehicles mainly used diesel as fuel, at the cost of 3.244 BRL per litre [16]. We also assume that trucks have an average consumption of 2.5 km/litre [17]. Finally, the unit shipping cost is posed as follows:

(2) $$c_{mn}^{\text{d}}=2\times c_{mn}^{\text{s}}=2\times \frac{\text{diesel}\;\text{cost}}{\text{consumption}}\cdot {\text{dist}}_{mn}$$

in which ${\text{dist}}_{mn}$ is the distance (in km) between two nodes.

Finally, prepositioning costs were estimated based on the most recent public announcement for procurement of humanitarian supplies by the Brazilian government [18]. These costs vary between regions. The holding cost of relief aid items $c_{r}^{\text{h}}$ was estimated as 10% of the cost of prepositioning relief aid items per year.

#### 2.2.4 Socioeconomic data

We used the data on income and size of poor populations as the poverty indicator parameter $b_{n}^{a}$. We obtained such indicators at the state-level from the same source [4] for the optimization instance (HL1). The parameters at the microregion-level were calculated using the weighted average by the population of each city for each microregion for the optimization instance (HL2). The reference income, i.e. minimum wage, can be used to assess the level of poverty in each node. For example, [1] used a FGT poverty measure that accounts for the difference between the income of poverty groups in relation to the reference income.

#### 2.2.5 Budget or emergency relief funds

We assumed that the budget for the preparedness phase η is restricted while the budget for the response phase $\eta^{\prime }$ is not. In order to establish the budget parameters according to [1], we first obtained the reference budgets for the first and second-stage operations via the problem of minimization of costs subject to the satisfaction of all victims’ needs. We then assumed that only 60% of the reference budget was available for the first-stage operations in the optimization instances (HL1 and HL2). Such assumptions are based on the small cost of the response phase in relation to overall costs. However, it is possible to obtain different instances by changing the budget values. For example, [1] varied the budget values to assess the sensitivity of the proposed optimization model to different budget restrictions. Also, the problem can be formulated in different manners that do not necessary require the budget parameters, if the aim of the study is to minimize the costs, or if other resources are limited, such as the available quantity of prepositioned relief aid items, for example.

#### 2.2.6 Remarks on the generation of real-world based instances

The data made available in this article can be used to generate real-world based instances. The new optimization instances will be dependent on the scope of locations under study (municipality, state, mesoregion, or microregion levels). As an example, we will briefly describe how the optimization instance (HL2) can be obtained via combination of some parameter values of the optimization instance (HL1) with partial data obtained from the presented datasets.

The optimization instance (HL2) represents a location-allocation problem that covers only the microregions of the state of Santa Catarina, while the instance (HL1) corresponds to the same problem at the national level, considering each state as a different node. For the generation of HL2, we first aggregated the number of victims for each year and the poverty indicators in Santa Catarina at the microregion level. We also calculated the distances between each microregion as shown in Section 2.2.2 (Distances), using the main municipality’s latitude and longitude of each microregion as input.

For the other parameters, we filtered the values available in HL1 that correspond to the Santa Catarina’s values, when applicable. For example, HL1 presents all states’ opening costs, including the opening costs for Santa Catarina. Then, we only considered the opening costs’ values of Santa Catarina, from HL1, in the optimization instance (HL2). We also kept all the other parameters that are common to both problems, such as the truck capacity and items’ volume, and then we updated the calculated parameters. Table 2 shows what parameters are kept, filtered or updated to generate a new optimization instance such as HL2, if no other external data is used.

**Table 2:** Parameters that are identical or changed in generated optimization instances, compared to HL1.

CAP_FAC	Z_COST	VICTIMS	R_DAYS	RHO	P_COST	DISTANCE	COVERAGE
=	∘	★	=	=	∘	★	=
P_INDICATOR	LENGHT	PI	FUEL	EFFICIENCY	LOAD	REFERENCE	BUD_1, BUD_2
★	=	=	=	=	=	=	★

= Identical data / ∘ Filtered data / ★ New data.

This procedure can be used to generate optimization instances for any scope of study that is available in the data table containing the number of disaster victims in Brazil. Following the steps mentioned above, it is possible to study specific areas and arrangements at different levels, using reliable data and realistic estimates that can help improving humanitarian supply chain design in Brazil. Even so, we encourage researchers to substitute the data provided here whenever they have access to a more accurate data or estimate. For example, if the researcher knows the prepositioning cost of relief aid items at the municipality level for a given state, they can use this data to substitute the prepositioning cost given in HL1 (state level) when carrying out experiments at the municipality level. The use of different instances is valuable for the development and validation of algorithms and optimization models. Researchers can take the steps mentioned in this section to generate a number of instances based on the Brazilian case that can support the performance evaluation of their own mathematical models.

## CRediT authorship contribution statement

**Rafaela Veloso:** Conceptualization, Methodology, Data curation, Writing – original draft. **Juliana Cespedes:** Validation, Writing – review & editing. **Aakil Caunhye:** Validation, Writing – review & editing. **Douglas Alem:** Validation, Writing – review & editing, Supervision.

## Declaration of Competing Interest

The authors declare that they have no known competing financial interests or personal relationships which have, or could be perceived to have, influenced the work reported in this article.

## Footnotes

4 GI is the Portuguese abbreviation for “Galpo Industrial” in the CUB reports, which was translated to “Industrial Shed” here.

## Footnotes

1 COBRADE is the Brazilian Classification and Coding of Disaster (*CodificaȺo Brasileira de Desastres* in Portuguese). Available at http://www.defesacivil.pr.gov.br/sites/defesa-civil/arquivos_restritos/files/documento/2018-12/Cobrade_comsimbologia.pdf.

2 A microregion is a subdivision of a mesoregion, defined according to the economic production and the spatial interaction between municipalities.

3 A mesoregion is a territorial subdivision proposed by the Brazilian Institute of Geography and Statistic (IBGE) that corresponds to a given set of municipalities in the same state, grouped according to the following criteria: social characteristics, geography and, spatial articulation.

## References

[1] Alem D., Veloso R., Bektas T., R Londe L. (2021). ‘Pro-poor’ humanitarian logistics: prioritizing the vulnerable in allocating relief aid. Optim. Online.

[2] Minister of Regional Development, Integrated Disaster Information System (S2ID) reports, 2021, (http://s2id.mi.gov.br). Accessed February 1, 2021.

[3] Brazilian Institute for Geography and Statistics (IBGE), Municipal Mesh, 2021, (https://www.ibge.gov.br/en/geosciences/territoriafl-organization/territorial-meshes.html). Accessed March 5, 2021.

[4] UNDP and Ipea, Atlas of Human Development in Brazil queries, 2013, (http://www.atlasbrasil.org.br/consulta/planilha. Accessed online 10 March 2021). Accessed July 18, 2021.

[5] Alem D., Bonilla-Londono H.F., Barbosa-Povoa A.P., Relvas S., Ferreira D., Moreno A. (2021). Building disaster preparedness and response capacity in humanitarian supply chains using the social vulnerability index. Eur. J. Oper. Res..

[6] Sabbaghtorkan M., Batta R., He Q. (2020). Prepositioning of assets and supplies in disaster operations management: review and research gap identification. Eur. J. Oper. Res..

[7] United Nations Development Programme (UNDP), Human Development report, 1990, (http://hdr.undp.org/sites/default/files/reports/219/hdr_1990_en_complete_nostats.pdf). Accessed March 13, 2021.

[8] Pinto D.G.C., Costa M.A.C., Marques M.L.d.A.C. (2003).

[9] Brazilian Institute for Geography and Statistics (IBGE), Population Estimates, 2021, (https://www.ibge.gov.br/en/statistics/social/population/). Accessed March 5, 2021.

[10] Bullock J., Haddow G. (2004).

[11] Kovács G., Spens K.M. (2007). Humanitarian logistics in disaster relief operations. Int. J. Phys. Distrib.Logist. Manage..

[12] Balcik B., Silvestri S., Rancourt M.-È., Laporte G. (2019). Collaborative prepositioning network design for regional disaster response. Prod. Oper. Manage..

[13] Gerdin M., Chataigner P., Tax L., Kubai A., von Schreeb J. (2014). Does need matter? Needs assessments and decision-making among major humanitarian health agencies. Disasters.

[14] OpenStreetMap, 2018, (https://www.openstreetmap.org). Accessed September 25, 2018.

[15] Brazilian Chamber of Construction Industry (CBIC), Basic Unit Costs of Construction reports, 2021, (http://www.cub.org.br/). Accessed April 15, 2021.

[16] National Agency for Petroleum, Natural Gas and Biofuels (ANP), Pricing Survey System technical report, 2021, (https://preco.anp.gov.br/). Accessed March 25, 2021.

[17] Brondani M., Hoffmann R., Mayer F.D., Kleinert J.S. (2015). Environmental and energy analysis of biodiesel production in Rio Grande do Sul, Brazil. Clean Technol. Environ. Policy.

[18] Minister of National Integration, Public Notice for Electronic Trading SRP n.09/17 - Humanitarian Assistance Kits, 2017, (http://www.integracao.gov.br/processo_licitatorio). Accessed September 25, 2018.

